# *CNTNAP2* variants affect early language development in the general population

**DOI:** 10.1111/j.1601-183X.2011.00684.x

**Published:** 2011-06

**Authors:** A J O Whitehouse, D V M Bishop, Q W Ang, C E Pennell, S E Fisher

**Affiliations:** † Telethon Institute for Child Health Research, Centre for Child Health ResearchPerth, Australia; ‡ Neurocognitive Development Unit, School of Psychology, University of Western AustraliaPerth, Australia; § Department of Experimental Psychology, University of OxfordOxford, United Kingdom; ¶ School of Women's and Infants' Health, University of Western AustraliaPerth, Australia; ** Wellcome Trust Centre for Human Genetics, University of OxfordOxford, United Kingdom; †† Language and Genetics Department, Max Planck Institute for PsycholinguisticsNijmegen, The Netherlands

**Keywords:** Autism, *CNTNAP2*, language delay, language development, Raine study, SLI

## Abstract

Early language development is known to be under genetic influence, but the genes affecting normal variation in the general population remain largely elusive. Recent studies of disorder reported that variants of the *CNTNAP2* gene are associated both with language deficits in specific language impairment (SLI) and with language delays in autism. We tested the hypothesis that these *CNTNAP2* variants affect communicative behavior, measured at 2 years of age in a large epidemiological sample, the Western Australian Pregnancy Cohort (Raine) Study. Singlepoint analyses of 1149 children (606 males and 543 females) revealed patterns of association which were strikingly reminiscent of those observed in previous investigations of impaired language, centered on the same genetic markers and with a consistent direction of effect (rs2710102, *P* = 0.0239; rs759178, *P* = 0.0248). On the basis of these findings, we performed analyses of four-marker haplotypes of rs2710102–rs759178–rs17236239–rs2538976 and identified significant association (haplotype TTAA, *P* = 0.049; haplotype GCAG, *P* = .0014). Our study suggests that common variants in the exon 13–15 region of *CNTNAP2* influence early language acquisition, as assessed at age 2, in the general population. We propose that these *CNTNAP2* variants increase susceptibility to SLI or autism when they occur together with other risk factors.

Although nearly all children learn to talk, there is substantial variation in the timing of language development. Around 10% of children can talk in sentences at 18 months of age, whereas the slowest 10% produce at most a handful of single words at this age (Neligan & Prudham 1969). Many late-talkers are actually ‘late bloomers', catching up with their peers by the time they are 3 or 4 years old (Thal & Katich 1997). Nevertheless, in some children late talking is the first indication of persistent language impairment (Haynes & Naidoo 1991) and in a minority of these it may be a symptom of autistic disorder (Hagberg *et al.* 2010).

It is often assumed that the age at which a child develops language is largely dependent on the language input he or she receives. However, a recent epidemiological study found that family history of delayed language development predicted late talking in 24-month-olds, while other factors, such as maternal education, birth risks and maternal depression, did not have significant influence (Zubrick *et al.* 2007). Data from twin studies indicate that inherited factors make substantial contributions to early language development (Dale *et al.* 1998) and affect levels of performance on components of language in the normal range of abilities (Kovas *et al.* 2005). Still, at this point very little is known regarding the specific genetic variants that are associated with language development in toddlers from the general population. Here, we address this issue through analyses of early communicative behavior in a large epidemiological sample.

Our investigations were tightly constrained by prior evidence from molecular studies of neurodevelopmental disorders, which have converged on *CNTNAP2* as a gene with relevance to language learning. One notable study reported associations between markers in *CNTNAP2* and parental report of ‘age at first word’ in probands with autism (Alarcón *et al.* 2008). Independent analyses of children with specific language impairment (SLI), but not autism, identified association of *CNTNAP2* variants with reduced performance on quantitative indices of language ability (Vernes *et al.* 2008). Intriguingly, these separate investigations of distinct language-related disorders (Whitehouse *et al.* 2007) highlighted the same markers and alleles within *CNTNAP2* as risk factors. *CNTNAP2* encodes a member of the neurexin superfamily – neuronal transmembrane proteins involved in cell adhesion – and shows enriched expression in language-related circuits of the brain (Abrahams *et al.* 2007). Moreover, this gene is directly regulated by FOXP2, a transcription factor mutated in rare monogenic forms of speech and language disorder (Fisher & Scharff 2009).

Thus, in the current investigation, we carried out a hypothesis-driven study of links between common *CNTNAP2* variants and early language proficiency, assessed at 24 months of age, in an epidemiological sample of over a thousand children (the Raine sample). We specifically targeted the same single-nucleotide polymorphisms (SNPs) across the *CNTNAP2* gene as those previously investigated in SLI by Vernes *et al.* (2008). Our hypothesis was that the particular *CNTNAP2* markers implicated in language impairments of SLI and delayed language in autism would extend their influence beyond disorder, to show association with early language acquisition in the general population.

## Materials and methods

### Participants

The Western Australian Pregnancy Cohort (Raine) Study is a longitudinal investigation of 2900 pregnant women and their offspring consecutively recruited from maternity units between 1989 and 1991 (Newnham *et al.* 1993). The inclusion criteria were (1) English language skills sufficient to understand the study demands, (2) an expectation to deliver at King Edward Memorial Hospital (KEMH) and (3) an intention to remain in Western Australia to enable future follow-up of their child. Ninety percent of eligible women agreed to participate in the study.

From the original cohort, 2868 children have been followed over two decades. Participant recruitment and all follow-ups of their families were approved by the Human Ethics Committee at King Edward Memorial Hospital and/or Princess Margaret Hospital for Children in Perth. The Raine sample is representative of the larger Australian population (88% Caucasian); only those children with both biological parents of White European origin were included in the current analyses. DNA and phenotypic data were available for 1149 children (606 males and 543 females).

### Phenotypic measure

Our study specifically concerned early indicators of language acquisition in toddlers, where direct assessment of ability can be challenging. For phenotyping at such young ages, parental report has been shown to provide a robust alternative to direct testing (Johnson *et al.* 2008). The Communication subscale of the Infant Monitoring Questionnaire (IMQ) (Bricker & Squires 1989) was administered when the child was 2 years old. This parent-completed checklist contains seven items assessing early communicative behavior, such as protoimperative actions (e.g. looking or pointing at an item to request it), the following of simple commands (e.g. ‘come here’, ‘sit down’), and the use of two- or three-word strings (e.g. ‘go, car’, ‘shut door’). Parents indicate whether their child shows this behavior always (2 points), sometimes (1 point) or never (zero points), yielding an overall score ranging from 0 to 14. The validity and reliability of the IMQ range from 0.85 to 0.9 (Bricker *et al.* 1988). Questionnaires with one missing item (*n* = 155) were prorated to yield a score out of 14. Scores were transformed from centile equivalents to *z*-scores to give a normally distributed variable.

### Genetic data

For the Raine study, DNA samples have been collected using standardized procedures at 14 or 16 years of age, followed by genotyping on an Illumina 660 Quad Array (San Diego, CA, USA). SNPs that did not meet quality control criteria (call rate ≥95%; minor allele frequency >0.05; Hardy–Weinberg disequilibrium *P* value >0.000001) were discarded. It is important to emphasize that, although genome-wide SNP data have been collected for this sample, we did not perform a hypothesis-free genome-wide association scan for our measure of interest. Instead, this study was a tightly constrained hypothesis-driven candidate gene approach, based on prior literature, which considered a set of 30 SNPs from the *CNTNAP2* gene [matching those from Vernes *et al.* (2008)]. This led us to a focused analysis of the rs2710102–rs759178–rs17236239–rs2538976 multimarker combination. No other markers from elsewhere in the genome were assessed for association with early communicative behavior in this sample.

### Data analysis

Our panel of 30 SNPs matching those used to study SLI in previous *CNTNAP2* analyses (Vernes *et al.* 2008) constituted the majority of the 38 SNPs assessed in the prior study. Each biallelic SNP was first tested for association with the quantitative measure of the communication phenotype using an allelic test of association within R (R Development Core Team 2009). On the basis of the previous findings by Vernes *et al.* (2008), our model assumed that the risk allele of the SNP had a dominant mode of action. Consideration of the singlepoint SNP findings, and their convergence with earlier studies, led us to test the four-marker haplotypes of rs2710102–rs759178–rs17236239–rs2538976, analyzing the three common alleles using R. Our analysis of each such multimarker allele involved two factors: (1) comparison between harboring two copies and one copy of the haplotype and (2) comparison between harboring two copies and no copies of the haplotype – allowing us to separately assess the modes of action of each of the three alleles. To minimize multiple testing, we did not analyze any further marker configurations. Linkage disequilibrium (LD) among *CNTNAP2* SNPs was determined with Haploview version 4.2 (http://www.broadinstitute.org/haploview/haploview) (Barrett *et al.* 2005). Haplotypes were inferred using SimHap version 1.0.2, and the most-likely haplotypes of each individual used as inputs for the R analyses described above.

Principal components analysis of genome-wide SNP data with Eigenstrat (Price *et al.* 2006) has revealed evidence of population stratification in the Raine sample, and so the first two principal components were included as cofactors in all analyses. This procedure has been used previously in genetic analyses of the Raine cohort (Paracchini *et al.* 2011).

## Results

We assessed the same panel of markers across *CNTNAP2* as Vernes *et al.* (2008), but focusing instead on a quantitative measure of early language in a general population cohort. This panel included most of the key SNPs that were significantly associated in that study, as well as the flanking markers from elsewhere in the gene that had not shown association. Our hypothesis was that a similarly localized subset of SNPs within the panel would show evidence of association in our sample, against a background of nonsignificant results. The pattern of single SNP associations in our general population sample (Table 1) was strikingly reminiscent of that observed by Vernes *et al.* (2008) in their SLI families, highlighting an almost identical subset of markers, located in the exon 13–15 region of *CNTNAP2*. Two neighboring SNPs – rs2710102 and rs759178 – showed nominal significance (*P* = 0.0239 and 0.0248) and another three markers in their vicinity – rs17236239, rs2538976 and rs2710117 – displayed suggestive trends (*P* values between 0.05 and 0.085). These markers corresponded to those showing strongest associations in the Vernes *et al.* (2008) study of SLI and overlapped with the most significant findings from the Alarcón *et al.* (2008) investigation of language delay in autistic probands. The effects observed were consistently in the same direction as prior studies; the alleles that correlated with reduced language performance in the Raine sample (Table 2) were the same as those identified as putative susceptibility alleles in studies of disorder [c.f. Table S3 in Vernes *et al.* (2008) and Table S1 in Alarcón *et al.* (2008)]. For example, risk alleles in SLI and autism were C for marker rs2710102 (C/T polymorphism) and G for marker rs759178 (G/T polymorphism); these same alleles were associated with lower early language scores in our general population sample (Table 2).

**Table 1:** Singlepoint association between *CNTNAP2* variants and a quantitative measure of early communicative behavior

SNP marker	Position (bp)*	Location	SNP†	MAF‡	*P* value§	SLI association¶
rs7806058	146007792	Intron 1	A/G	0.35	ns	−
rs6946112	146059217	Intron 1	C/T	0.27	ns	−
rs12703803	146062909	Intron 1	T/G	0.29	ns	−
rs2058377	146090070	Intron 1	A/G	0.31	ns	−
rs12667234	146111176	Intron 1	A/G	0.30	ns	−
rs2888335	146124574	Intron 1	T/C	0.30	ns	−
rs7805539	146160278	Intron 1	G/A	0.28	ns	−
rs4726793	146276890	Intron 1	A/G	0.20	ns	−
rs10277654	146352576	Intron 1	T/C	0.44	ns	−
rs7794745	146489606	Intron 2	A/T	0.26	ns	−
rs6945085	146691220	Intron 3	T/C	0.09	ns	−
rs1024676	146715861	Intron 3	C/T	0.38	ns	−
rs10282158	146738067	Intron 3	T/A	0.06	ns	−
rs7812091	146740577	Intron 3	T/C	0.38	ns	−
rs10500170	146848251	Intron 8	A/G	0.16	ns	−
rs1603453	146908919	Intron 8	T/A	0.11	ns	−
rs1603450	146913540	Intron 8	G/A	0.18	0.0426*	−
rs10251377	147117454	Intron 10	A/G	0.25	ns	−
rs851715	147526906	Intron 13	A/G	0.32	ns	+
rs1177007	147546371	Intron 13	A/G	0.31	ns	−
rs10246256	147554807	Intron 13	T/C	0.31	ns	+
rs2710102	147574390	Intron 13	C/T	0.49	0.0239*	+
rs759178	147575112	Intron 13	G/T	0.49	0.0248*	+
rs17236239	147582305	Intron 13	A/G	0.35	0.0851	+
rs2538976	147585819	Intron 13	G/A	0.50	0.0535	+
rs2538963	147599446	Intron 13	G/A	0.32	ns	−
rs2710117	147601772	Intron 14	A/T	0.38	0.0771	+
rs10240503	147674978	Exon 15	A/G	0.11	ns	−
rs12155129	147856865	Intron 17	A/G	0.06	ns	−
rs11980146	147956733	Intron 20	A/G	0.34	ns	−

ns, not significant.

* Position based on the hg19 assembly of the Human Genome sequence.

† Alleles of each SNP are given with respect to the forward strand of chromosome 7.

‡ Minor allele frequency within the Raine sample.

§ *P* values <0.1 are shown, with *P* values <0.05 denoted by an asterisk.

¶ Summary of findings from the Vernes *et al.* (2008) study of SLI: ‘+’ indicates SNPs showing significant association in that study, whereas ‘−’ denotes negative results.

**Table 2:** Effects of singlepoint *CNTNAP2* variants on early communicative behavior

		Putative risk allele				
					
SNP marker	SNP*	Raine†	SLI‡	Non-risk homozygote§	Heterozygote§	Risk homozygote§
rs1603450	G/A	G	na	0.649 (0.841)	0.204 (0.961)	0.221 (0.958)
rs2710102	C/T	C	C	0.356 (0.977)	0.189 (0.958)	0.191 (0.933)
rs759178	G/T	G	G	0.356 (0.977)	0.191 (0.958)	0.190 (0.935)
rs17236239	A/G	G	G	0.293 (0.969)	0.166 (0.959)	0.246 (0.907)
rs2538976	G/A	G	G	0.336 (0.972)	0.201 (0.954)	0.180 (0.949)
rs2710117	A/T	A	A	0.368 (0.954)	0.228 (0.966)	0.186 (0.937)

na, not applicable.

* Alleles of each SNP are given with respect to the forward strand of chromosome 7.

† Allele which was correlated with reduced scores in the Raine sample.

‡ Allele which was correlated with reduced scores in the Vernes *et al.* (2008) study of SLI.

§ Mean (and SD) scores of the language phenotype at age 2 years (*z*-score transformed scores on the Communication subscale of the Infant Monitoring Questionnaire) according to diploid genotype in the Raine sample.

In the main cluster of associated SNPs – rs2710102, rs759178, rs17236239, rs2538976 – the markers were in strong LD, with D′ values of 1 for all pairwise comparisons (Figure S1, Supporting information). Notably, these four SNPs were central to a nine-marker risk haplotype previously studied by Vernes *et al.* (2008). We therefore constructed multimarker haplotypes using these four neighboring SNPs and identified three common combinations (TTAA, CGGG and CGAG), representing 98% of individuals (Table 3). As expected from the direction of effects observed in the singlepoint results (Table 2) and consistent with prior published results (Vernes *et al.* 2008), the TTAA multimarker allele was associated with higher scores on the measure of early language, whereas the CGGG and CGAG alleles were associated with reduced scores. TTAA showed nominal significance (*P* = 0.0488) and CGGG displayed a suggestive trend (*P* = 0.0627), but the strongest association was for CGAG (*P* = 0.0014); this remains significant after accounting for the number of tests that we performed in the study (30 singlepoint tests and 3 haplotypic analyses). Children carrying two copies of this haplotype obtained substantially lower scores (mean = −0.355, SE = 0.169) than those with one copy (mean 0.313, SE = 0.055) or no copies (mean = 0.223, SE = 0.033).

**Table 3:** Association of rs2710102–rs759178–rs17236239–rs2538976 haplotypes with a quantitative measure of early communicative behavior

Haplotype*	Frequency†	*P* value	Factor‡
TTAA	0.48	0.0488	2
CGGG	0.35	0.0627	1
CGAG	0.15	0.0014	2

* Alleles are given with respect to the forward strand of chromosome 7.

† Frequency of haplotype within the Raine sample.

‡ Analysis in R assessed two factors: 1 = comparison between harboring two copies and one copy of the haplotype; 2 = comparison between harboring two copies and no copies of the haplotype. This column indicates which factor yielded the most significant result, as reported in the preceding column.

## Discussion

Our results suggest that variants in the exon 13–15 region of *CNTNAP2* previously associated with deficits in SLI (Vernes *et al.* 2008) and delayed language in autism (Alarcón *et al.* 2008; Poot *et al.* 2010) also affect the early stages of language development in children from the general population. This was a targeted hypothesis-driven study of a single gene, focusing on specific markers that have been strongly implicated in multiple prior reports of language-related disorder, rather than a genome-wide search for new variants.

The consistencies in findings across multiple investigations are noteworthy given several key differences in the natures of these studies. Alarcón *et al.* (2008) studied probands with autism in an American sample, employing a parental report of language delay. Vernes *et al.* (2008) assessed a UK sample, examined language test scores in older children and focused on families selected for SLI. In this study, we investigated an Australian sample, used a parental report measure assessing language development at age 2, and tested for association across the normal range. Despite the obvious differences in sample ascertainment and phenotypic characterization, there was agreement not only regarding the pattern of SNPs that were associated but also in the direction of allelic effects.

In our study, we constructed a single set of haplotypes using four neighboring markers in high LD which, based on the singlepoint pattern of results, appeared to form a core site of association. Although we did not genotype every associated marker from the Vernes *et al.* (2008) study, these four markers were central to the nine-marker haplotypes that they previously assessed in SLI. Thus, our haplotypic alleles would be expected to capture much of the relevant variation from the earlier investigation. Indeed, haplotypic analyses from the two studies are generally concordant – both investigations found that the TTAA multimarker allele of rs2710102–rs759178–rs17236239–rs2538976 is associated with higher scores, whereas the alternative CGGG/CGAG alleles are associated with reduced performance (c.f. Table S4 of Vernes *et al.* 2008). However, although the CGGG allele showed the strongest association in the SLI study, our analyses of the Raine sample identified much more significant effects for the rare CGAG combination, which here had particularly dramatic effects on language scores. These differences in haplotypic background could relate to the distinct population history of the samples. Regardless, the data suggest that in the vicinity of rs2710102–rs759178–rs17236239–rs2538976 there lie specific functional risk variants (as yet unidentified) with particular relevance to early language acquisition. Of note, the *CNTNAP2* gene locus is one of the largest in the genome and could potentially contain multiple additional sites with functional relevance to neurodevelopmental phenotypes, to be clarified in future with high-density SNP screening and sequence-based strategies.

A methodological conclusion from our study is that a simple parental questionnaire focused on early language development can provide valuable phenotypic information for molecular genetic analyses, which may be particularly pertinent given the difficulties in directly assessing a child's performance in the earliest years of life. This is consistent with the core findings of Alarcón *et al.* (2008), who reported that rs2710102 and neighboring variants were associated with just a single item from the Autism Diagnostic Inventory – Revised (Lord *et al.* 1994),‘age at first word’, in autistic probands. In addition, in a recent study of multiple traits contributing to the autistic spectrum, Steer *et al.* (2010) reported a nominal association between rs17236239 and a factor they termed ‘language acquisition’, which primarily loaded on parental report measures of early language development. Our conclusion is also in line with the findings of Johnson *et al.* (2008), who showed good agreement between parent report and direct assessment of children's abilities at 2 years of age.

In terms of theoretical implications, it is clear that these common *CNTNAP2* variants are not sufficient by themselves to account for language and communication disorders in children. This conclusion is in line with the current consensus that both SLI and autism are complex disorders resulting from the combined effect of multiple influences (Geschwind 2008). We hypothesize that *CNTNAP2* variants which usually yield only a small boost or lag in language acquisition will have more marked consequences when they occur in concert with other genetic or environmental risk factors. Bishop (2010) suggests that autism may result from epistatic rather than additive interactions between genes. From this perspective, it would be of considerable interest to see whether there are additive or interactive effects of *CNTNAP2* with genetic variants affecting social cognition, such as a recently described locus on chromosome 5p14 (St Pourcain *et al.* 2010).

## Supporting Information

**Figure S1:** Location and linkage disequilibrium of 30 SNPs on the *CNTNAP2* gene. The top of the figure provides an indication of the genomic location of each SNP on chromosome 7q. In total, 30 SNPs were analyzed across a 2000-kb interval. Black lines indicate the position of each SNP within *CNTNAP2*. Inter-SNP linkage disequilibrium was generated with Haploview. The upper panel reports D′ values within cells. Empty red cells represent full LD and empty blue cells represent lack of LD. The lower panel reports *r*^2^ values within cells. Empty white cells represent lack of LD and darker shading represent increasingly stronger LD. Haploview identified five LD blocks (black solid lines) using the confidence interval method (Gabriel et al. 2002).

As a service to our authors and readers, this journal provides supporting information supplied by the authors. Such materials are peer-reviewed and may be re-organized for online delivery, but are not copy-edited or typeset. Technical support issues arising from supporting information (other than missing files) should be addressed to the authors.
