# Supplementary material for: CNTNAP2 variants affect early language development in the general population
Source: Genes Brain Behav. 2011 Jun;10(4):451–6. doi: 10.1111/j.1601-183X.2011.00684.x (PMC3130139; doi:10.1111/j.1601-183X.2011.00684.x)
Supplement: Supplementary file 1 [file gbb0010-0451-SD1.doc]

**Figure S1.** **Location and Linkage Disequilibrium of 30 SNPs on the *CNTNAP2* gene.** The top of the figure provides an indication of the genomic location of each SNP on chromosome 7q. In total, 30 SNPs were analysed across a 2000 kb interval. Black lines indicate the position of each SNP within *CNTNAP2*. Inter-SNP linkage disequilibrium was generated with Haploview. The upper panel reports D’ values within cells. Empty red cells represent full LD and empty blue cells represent lack of LD. The lower panel reports *r*^2^ values within cells. Empty white cells represent lack of LD and darker shading represent increasingly stronger LD. Haploview identified five LD blocks (black solid lines) using the Confidence Interval method (Gabri*el et a*l., 2002).

Gabriel, S.B., Schaffner, S.F., Nguyen, H., Moore, J.M., Roy, J., Blumenstiel, B., Higgins, J., DeFelice, M., Lochner, A., Faggart, M., Liu-Cordero, S.N., Rotimi, C., Adeyemo, A., Cooper, R., Ward, R., Lander, E.S., Daly, M.J. & Altshuler, D. (2002) The structure of haplotype blocks in the human genome. *Science,* **296,** 2225-2229.


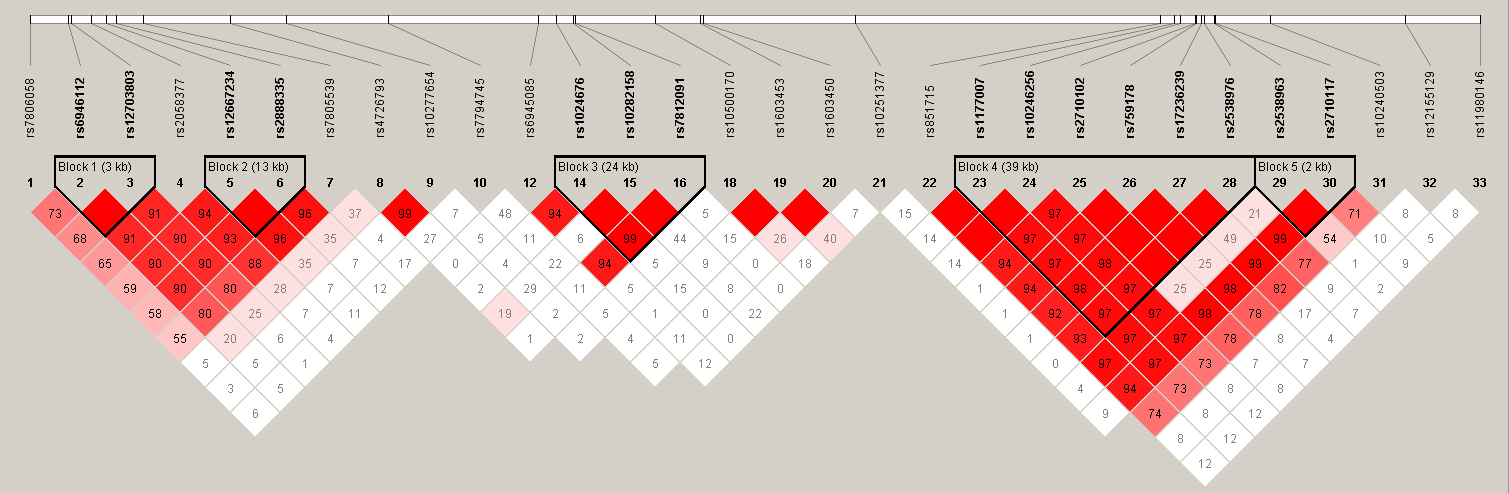


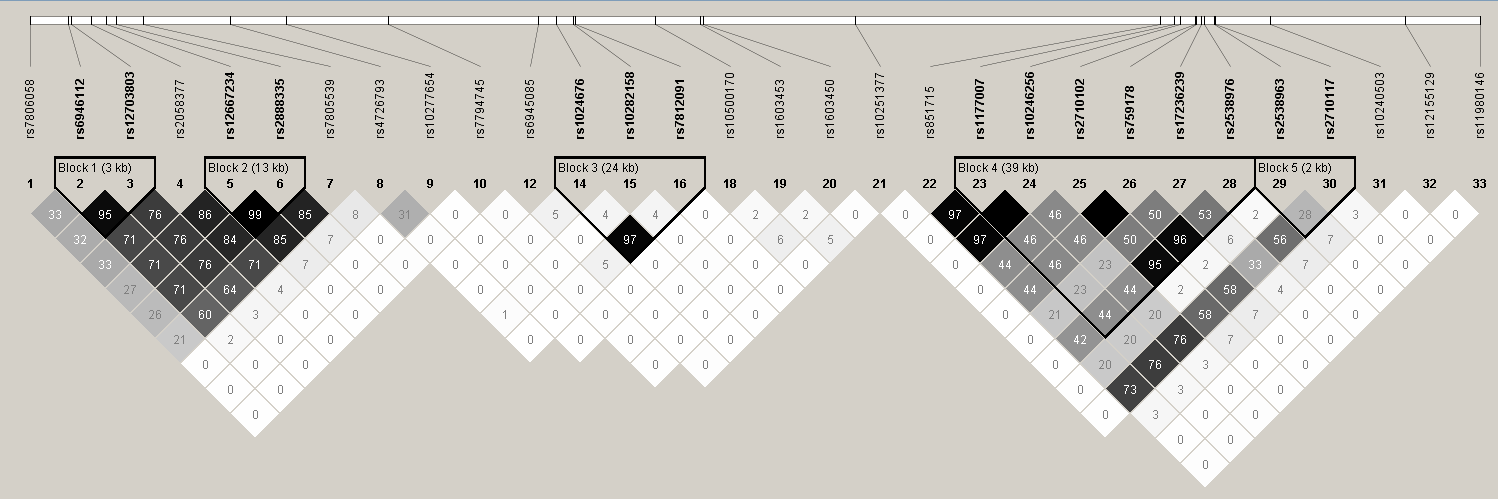


146000K 146400K 146800K 147200K 147600K 148000K
